# Overexpression of GRK2 in vascular smooth muscle leads to inappropriate hypertension and acute heart failure as in clinical scenario 1

**DOI:** 10.1038/s41598-023-34209-5

**Published:** 2023-05-12

**Authors:** Hiroki Yano, Kenji Onoue, Shiho Tokinaga, Tomoko Ioka, Satomi Ishihara, Yukihiro Hashimoto, Yasuki Nakada, Hitoshi Nakagawa, Tomoya Ueda, Ayako Seno, Taku Nishida, Makoto Watanabe, Yoshihiko Saito

**Affiliations:** Department of Cardiovascular Medicine, Nara Medical University, 840 Shijocho, Kashihara, Nara 634-8522 Japan

**Keywords:** Cardiology, Pathogenesis

## Abstract

Clinical scenario 1 (CS1) is acute heart failure (HF) characterized by transient systolic blood pressure (SBP) elevation and pulmonary congestion. Although it is managed by vasodilators, the molecular mechanism remains unclear. The sympathetic nervous system plays a key role in HF, and desensitization of cardiac β-adrenergic receptor (AR) signaling due to G protein-coupled receptor kinase 2 (GRK2) upregulation is known. However, vascular β-AR signaling that regulates cardiac afterload remains unelucidated in HF. We hypothesized that upregulation of vascular GRK2 leads to pathological conditions similar to CS1. GRK2 was overexpressed in vascular smooth muscle (VSM) of normal adult male mice by peritoneally injected adeno-associated viral vectors driven by the myosin heavy chain 11 promoter. Upregulation of GRK2 in VSM of GRK2 overexpressing mice augmented the absolute increase in SBP (+ 22.5 ± 4.3 mmHg vs. + 36.0 ± 4.0 mmHg, P < 0.01) and lung wet weight (4.28 ± 0.05 mg/g vs. 4.76 ± 0.15 mg/g, P < 0.01) by epinephrine as compared to those in control mice. Additionally, the expression of brain natriuretic peptide mRNA was doubled in GRK2 overexpressing mice as compared to that in control mice (P < 0.05). These findings were similar to CS1. GRK2 overexpression in VSM may cause inappropriate hypertension and HF, as in CS1.

## Introduction

Heart failure (HF), a debilitating syndrome with complex pathogenesis and clinical manifestations, has become a major health problem in recent years^1^. A subgroup of HF patients are clinically characterized by transient and rapid elevation of systolic blood pressure (SBP), which is inappropriate for heart function and causes subsequent pulmonary congestion without substantial weight gain on account of systemic congestion^2–6^. The clinical manifestations demonstrated by this subgroup are collectively referred to as clinical scenario 1 (CS1)^7^, and are usually alleviated by vasodilator administration. However, the underlying molecular mechanisms responsible for the same are poorly understood.

Research in the past three decades has led to a better understanding of the sympathetic nervous system which plays a crucial role in the development and worsening of HF^8–11^. Cardiac β-adrenergic receptor (AR) downregulation in conjunction with upregulation of G protein-coupled receptor kinase 2 (GRK2), which participates in desensitization or uncoupling of β-AR signaling has been investigated in depth^12–14^. In contrast, alteration of the β-AR signaling in the vasculature in HF has not been well understood, besides vascular constriction and dilation regulate afterload for the heart^4,6^.

Activation of β-AR signaling induces positive inotropic and chronotropic effects in the heart along with dilation of vasculature, leading to increased cardiac output^15,16^. Further, the activation of the sympathetic nervous system in acute HF is accompanied by elevation of serum catecholamine levels^8–11^. We therefore hypothesized that dysfunctional vascular β-AR signaling as evidenced by desensitization of the same due to elevation in circulating catecholamine levels, which possibly suppresses vasodilatory responses leading to excessive elevation of blood pressure along with pulmonary congestion, may be implicated in acute HF as seen in CS1.

Here we show exaggerated SBP elevation post epinephrine administration and subsequent pathological findings similar to HF using mice overexpressing GRK2 in the vessels generated by intraperitoneal injection of adeno-associated virus (AAV) vectors under the control of *myosin heavy chain 11* (*Myh11*), accompanied by induction of β-AR signaling uncoupling.

## Results

### Delivery of AAV vectors to vascular smooth muscle

**Figure 1:**
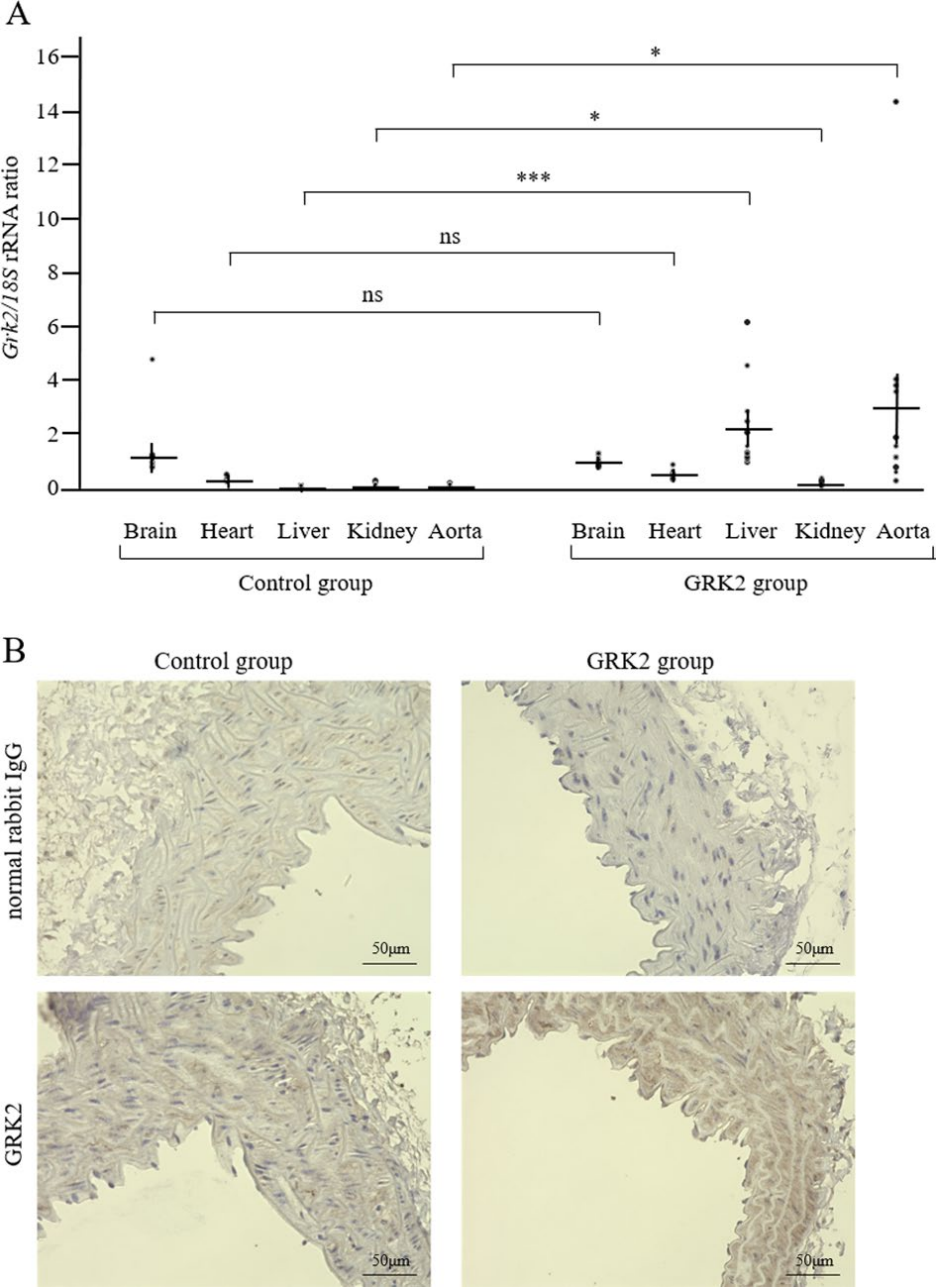
Expression of the GRK2 transgene. (**A**) Expression of the *Grk2* transgene was determined by real-time PCR in the control (n = 12) and GRK2 groups (n = 11). (**B**) Representative immunohistochemical staining images of in vivo protein GRK2 levels in aortas from mice in both groups. Results are expressed as mean ± SEM. *GRK2* G protein-coupled receptor kinase 2, *rRNA* ribosomal RNA, *ns* not significant. *P < 0.05, ***P < 0.001.

Tissue samples were collected post 8-weeks of AAV infection, followed by real-time polymerase chain reaction (PCR) to assess *Grk2* mRNA expression levels. Substantially higher relative expression levels of *Grk2* mRNA were evident in the aortas of the GRK2 group (2.97 ± 1.20, n = 11) as compared to those in the control group (0.05 ± 0.01, n = 12) (P = 0.019, Fig. 1A). Notably, a slight increase in the expression of *Grk2* mRNA was observed in the livers of the GRK2 group (2.27 ± 0.50, n = 11) in comparison to that in the livers of the control group (0.04 ± 0.01, n = 12) (P < 0.01, Fig. 1A). All other organs tested, including the brain, heart, and kidney demonstrated lower *Grk2* mRNA expression in both the control and GRK2 groups as compared to that in the aorta of the GRK2 group though relative expression levels of *Grk2* mRNA in the kidney were statistically higher in the GRK2 group (0.19 ± 0.02, n = 11) as compared to those in the control group (0.11 ± 0.02, n = 12) (P = 0.013, Fig. 1A). Immunohistochemical assessment of aortic GRK2 protein expression confirmed that the AAV infection technique utilized by us allowed for virus-mediated protein expression in the vascular smooth muscle (VSM) layer. This was evident from the strong GRK2 staining in the aortic VSM layer of AAV-DJ8-GRK2 infected mice in the representative image in Fig. 1B. In contrast, a weaker GRK2 signal was detected in the control AAV-DJ8-green fluorescent protein (GFP) infected aorta. GRK2 protein expression analyses in other organs mirrored the findings on *Grk2* mRNA expression levels. Notably, the GRK2 signal was almost undetectable in the brain, heart, and kidney tissues of both the control and GRK2 groups, and a slightly stronger GRK2 signal in the GRK2 group in comparison to that in the control group was only evident in the liver. There were no obvious fibrotic changes in the liver of both the control and GRK2 groups, which were assessed histologically with Masson’s trichrome stain (data not shown).

### Baseline blood pressure and heart rate

**Figure 2:**
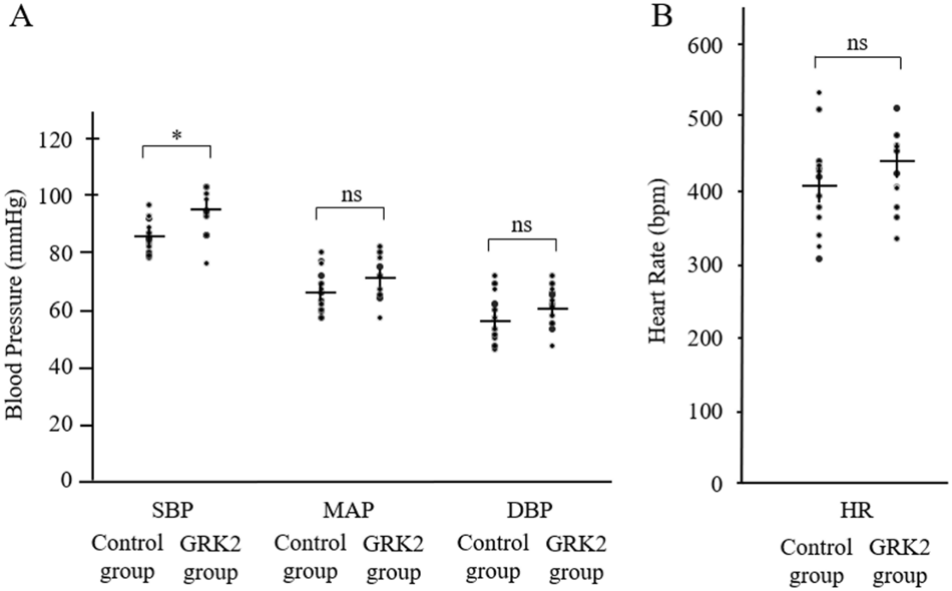
Baseline blood pressure and heart rate. (**A**) and (**B**) Baseline resting SBP, MAP, DBP and HR in the control (n = 13) and GRK2 (n = 12) groups. Results are expressed as mean ± SEM. Comparisons of parameters were performed using the 2-tailed Student *t* test or Welch test. *DBP* diastolic blood pressure, *GRK2* G protein-coupled receptor kinase 2, *HR* heart rate, *MAP* mean arterial pressure, *SBP* systolic blood pressure, *ns* not significant. *P < 0.05.

The effect of GRK2 on vascular function in vivo was evaluated by measuring blood pressure (BP) in mice with an indwelling carotid artery catheter. SBP was increased in the GRK2 group (93.8 ± 2.5 mmHg, n = 12) as compared to that in the control group (86.3 ± 1.7 mmHg, n = 13) (P = 0.020, Fig. 2A). Mean arterial pressure (MAP), diastolic blood pressure (DBP), and heart rate (HR) did not differ significantly between the control and GRK2 groups (Fig. 2A,B).

### BP and HR after epinephrine injection

**Figure 3:**
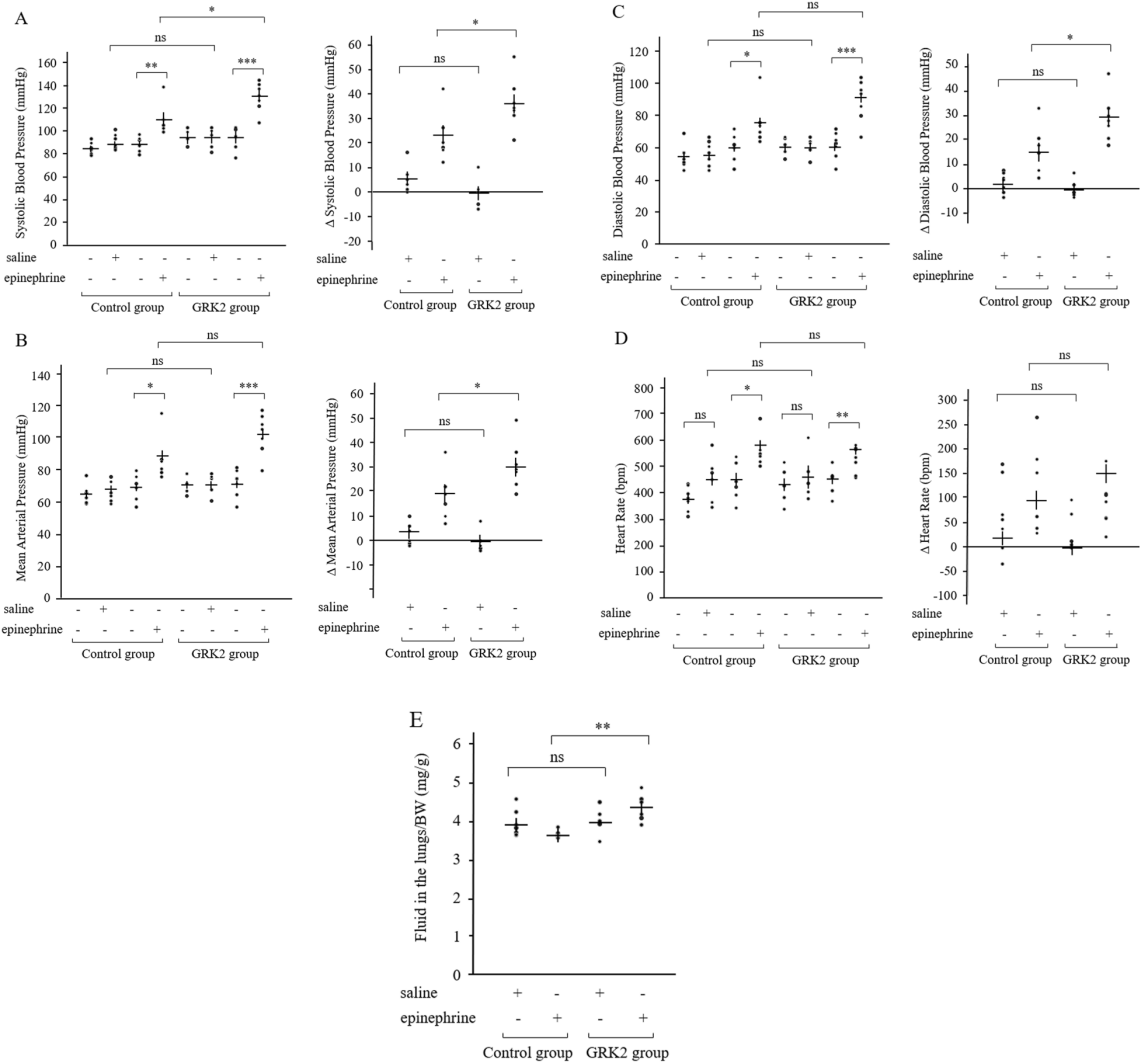
Blood pressure and heart rate pre and post either epinephrine or saline injection. SBP (**A**), MAP (**B**), DBP (**C**) and HR (**D**) in the control (n = 13) and GRK2 (n = 12) groups. (**E**) Ratio of pulmonary edema to body weight (Fluid in the lungs/BW) in the control (n = 12) and GRK2 (n = 11) groups. Results are expressed as mean ± SEM. Comparisons of parameters were performed using either the 2-tailed Student *t* test or Welch test, and repeated-measures 2-way ANOVA was used for multiple comparisons. *BW* body weight, *DBP* diastolic blood pressure, *GRK2* G protein-coupled receptor kinase 2, *HR* heart rate, *MAP* mean arterial pressure, *rRNA* ribosomal RNA, *SBP* systolic blood pressure, *ns* not significant. *P < 0.05, **P < 0.01, ***P < 0.001.

The effect of vascular GRK2 post epinephrine injection was assessed by measuring in vivo BP changes in mice. As seen in Fig. 3A, SBP significantly increased post epinephrine injection as compared to that prior to drug administration in the control and GRK2 groups. Further, SBP post epinephrine injection was significantly higher in the GRK2 group (130.0 ± 4.9 mmHg, n = 7) as compared to that in the control group (110.8 ± 5.9 mmHg, n = 6) (P = 0.028). The absolute increase in SBP seen after epinephrine administration was also significantly higher in the GRK2 group (+ 36.0 ± 4.0 mmHg, n = 7) in comparison to that in the control group (+ 22.5 ± 4.3 mmHg, n = 6) (P < 0.01, Fig. 3A). Additionally, a significant correlation between the relative expression levels of *Grk2* mRNA and SBP changes post epinephrine injection was clearly apparent (Supplementary Fig. S1). Significant elevations in MAP and DBP were also observed post epinephrine injection in comparison to that prior to drug administration in the control and GRK2 groups (Fig. 3B,C). However, MAP and DBP post epinephrine injection were not found to be significantly different between the GRK2 and control groups. The absolute increase in MAP seen after epinephrine injection was found to be higher in the GRK2 group (+ 30.7 ± 3.7 mmHg, n = 7) as compared to that in the control group (+ 18.2 ± 4.2 mmHg, n = 6) (P = 0.046, Fig. 3B). Similarly, the absolute increase in DBP seen after epinephrine injection was also higher in the GRK2 group (+ 28.0 ± 3.6 mmHg, n = 7) as compared to that in the control group (+ 15.7 ± 4.1 mmHg, n = 6) (P = 0.043, Fig. 3C). In contrast, no significant differences in SBP, MAP, and DBP post administration of a similar quantity of saline was observed between the control and GRK2 groups (Fig. 3A–C). Further, while mice HR was found to be significantly elevated by epinephrine administration in the control and GRK2 groups, it did not differ significantly between the two groups (Fig. 3D).

### Variable parameters related to the development of acute HF

**Figure 4:**
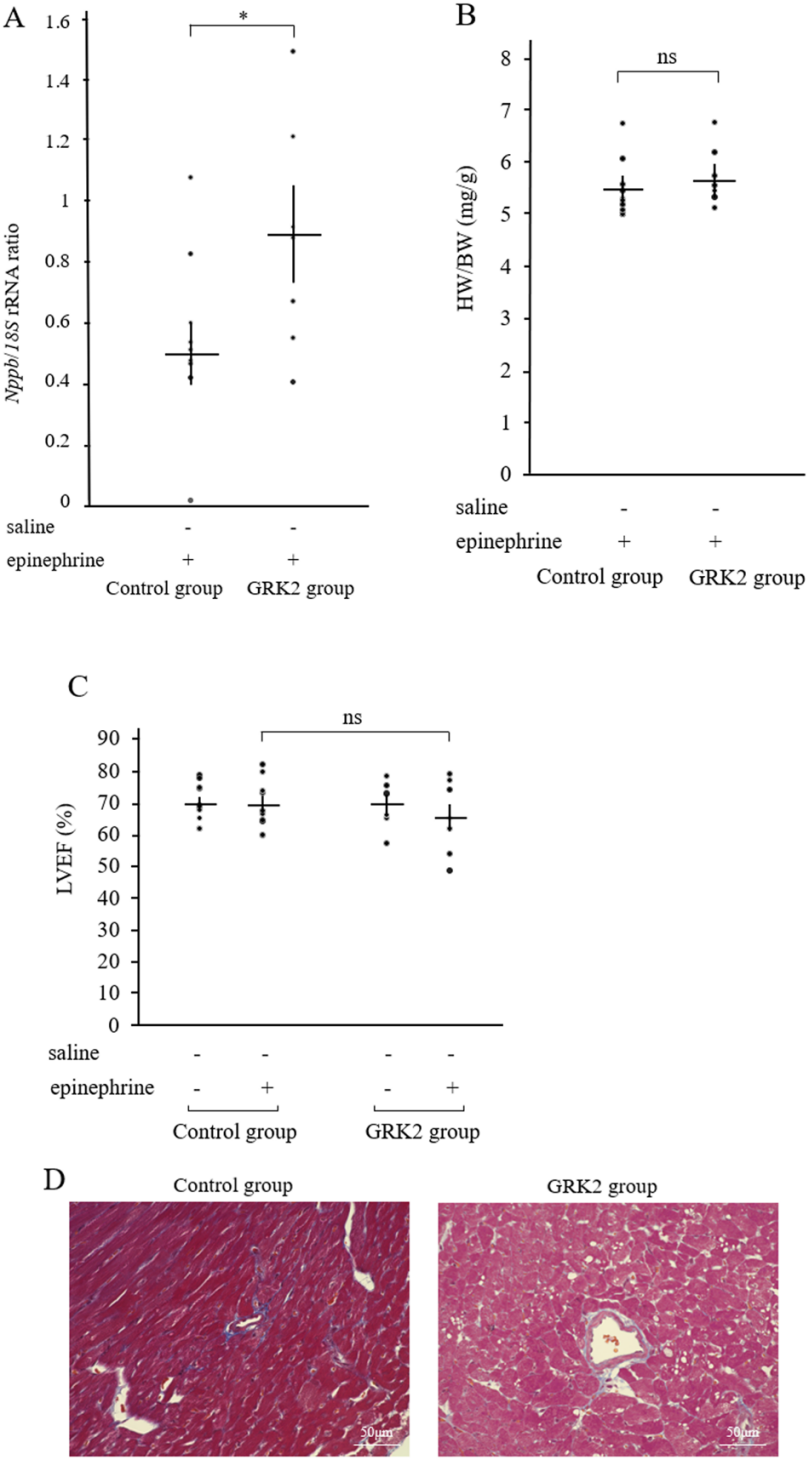
Elevation of *Nppb* and LVEF was preserved in GRK2 overexpressing mice after epinephrine injection. (**A**) Left ventricular *Nppb* mRNA expression levels in the control (n = 10) and GRK2 (n = 7) groups. (**B**) Ratio of heart weight to body weight (HW/BW) in the control (n = 10) and GRK2 (n = 7) groups. (**C**) Percentage of LVEF in the control (n = 10) and GRK2 (n = 7) groups. (**D**) Representative Masson’s trichrome stain images in small coronary arteries in the myocardium of the left ventricle from mice in both groups. Results are expressed as mean ± SEM. Comparisons of parameters were performed using the 2-tailed Student *t* test or Welch test. *Nppb natriuretic peptide B*, *BW* body weight, *GRK2* G protein-coupled receptor kinase 2, *HW* heart weight, *LVEF* left ventricular ejection fraction, *rRNA* ribosomal RNA, *ns* not significant. *P < 0.05.

Pulmonary fluid weight (the difference in weight before and after drying) was found to increase post intraperitoneal injection of epinephrine in the GRK2 group (4.76 ± 0.15 mg/g, n = 6) as compared to that in the control group (4.28 ± 0.05 mg/g, n = 5) (P < 0.01, Fig. 3E). In contrast, intraperitoneal injection of a similar quantity of saline had no significant difference on the pulmonary fluid weight between the control and GRK2 groups (Fig. 3E). Additionally, the relative expression levels of ventricular *natriuretic peptide B* (*Nppb*) mRNA post epinephrine injection significantly increased in the GRK2 group (0.87 ± 0.15, n = 7) as compared to that in the control group (0.49 ± 0.10, n = 10) (P = 0.042, Fig. 4A). There was not a significant correlation, but the expression levels of *Nppb* mRNA post epinephrine injection was tended to increase with *Grk2* mRNA expression levels (Supplementary Fig. S2). Heart weight after 8 weeks of AAV injection was not found to differ significantly between the control and GRK2 groups (Fig. 4B). Further, no significant differences were observed in the left ventricular ejection fraction (LVEF) before and after epinephrine injection between the control and GRK2 groups (Fig. 4C). Regarding the small coronary artery in the myocardium of the left ventricle, there were no obvious peri-vascular fibrotic changes of both the control and GRK2 groups, which were assessed histologically with Masson’s trichrome stain (Fig. 4D).

### β-AR uncoupling in VSM

**Figure 5:**
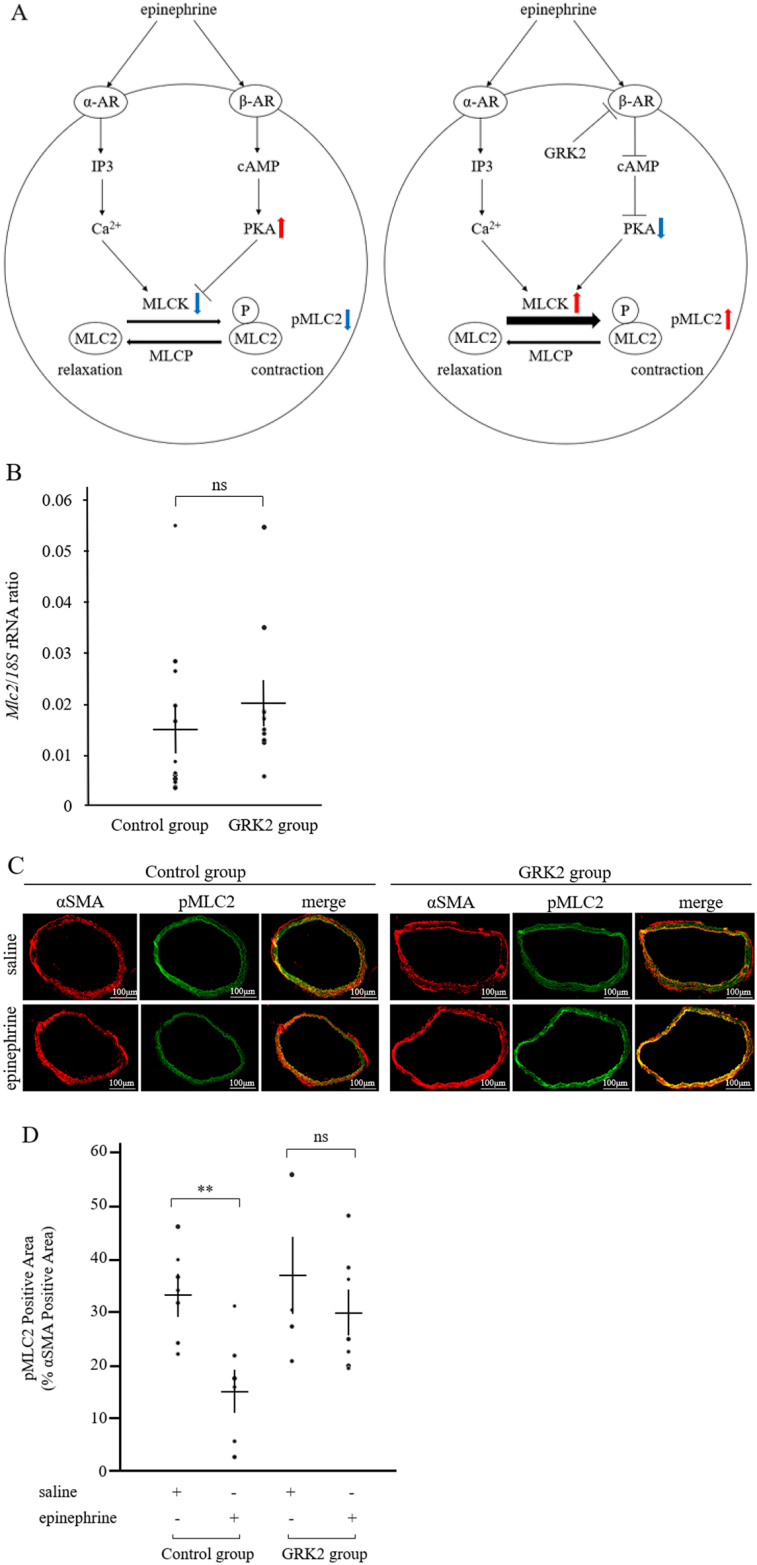
GRK2 overexpression inhibited vasodilation at the molecular level. The control (n = 13) and the GRK2 (n = 12) groups were exposed to saline or epinephrine respectively. (**A**) Schematic representation of the underlying mechanism of VSM contraction induced by epinephrine administration in the control and GRK2 groups. (**B**) Expression of the *Mlc2* transgene determined by real-time PCR. (**C**) Representative images of pMLC2 immunofluorescent staining in the descending aorta. Scale bar = 100 μm. (**D**) Quantification of pMLC2 positive areas. Data are represented as means ± SEM. *α-AR* alpha-adrenergic receptor, *β-AR* beta-adrenergic receptor, *cAMP* cyclic adenosine monophosphate, *GRK2* G protein-coupled receptor kinase 2, *IP3* inositol triphosphate, *MLC2* myosin light chain 2, *MLCK* myosin light chain kinase, *MLCP* myosin light chain phosphatase, *PKA* protein kinase A, *pMLC2* phosphorylated myosin light chain 2, *rRNA* ribosomal RNA, *VSM* vascular smooth muscle, *ns* not significant. **P < 0.01.

The effect of enhanced GRK2 expression on β-AR signaling in VSM was assessed by examining intracellular phospho-myosin light chain 2 (pMLC2) accumulation. The pMLC2 levels are found to be downregulated via protein kinase A (PKA) activation subsequent to β-AR stimulation (Fig. 5A). No significant differences were observed in the total *Mlc2* mRNA expression levels between the control and GRK2 groups (Fig. 5B). Further, the pMLC2:αSMA ratio (pMLC2/αSMA) was decreased post epinephrine treatment (15.6 ± 3.4%, n = 6) as compared to that observed post receipt of saline (33.2 ± 3.1%, n = 7, P < 0.01) in the control group (Fig. 5C,D). In contrast, a similar effect was not observed consequent to epinephrine treatment in the GRK2 group (epinephrine, 29.6 ± 4.1%, n = 7 vs. saline, 37.7 ± 6.9%, n = 5, P = 0.33), which was not inconsistent with PKA inactivation subsequent to β-AR desensitization (Fig. 5A).

## Discussion

The present study demonstrates that GRK2 overexpression in VSM augmented SBP elevation and pulmonary congestion as indicated by increased lung weight along with upregulation of ventricular *Nppb* mRNA expression induced by epinephrine injection, which are accompanied with attenuation of vascular β-AR signaling.

PKA activation is known to phosphorylate myosin light chain kinase (MLCK) post β-AR stimulation in VSM. Subsequently, phosphorylated MLCK self inactivates leading to downregulation of pMLC2 that result in vasodilation^17,18^. As shown in Fig. 5C, pMLC2 was observed to accumulate in the VSM of GRK2 overexpressing mice, which is suggestive of significant β-AR signaling desensitization by epinephrine. Notably, various cardiovascular effects via α-AR and β-AR signaling are exerted by epinephrine. The drug is known to increase cardiac output by exerting inotropic and chronotropic effects on the heart. Additionally, it has a vasodilatory effect via downregulation of pMLC2 downstream of PKA, and vasoconstrictive effect that is exerted via α-AR signaling^19^. All three types of β-ARs, namely β1, β2, and β3 mediate vascular relaxation. Of these, while β1- and β2-ARs can be desensitized by GRK2, β3-AR, a non-dominant blood vessel β-AR is resistant to GRK2 mediated phosphorylation^20–23^. Eckhart et al. reported that while GRK2 phosphorylates α1-AR in vitro, the receptor is refractory to modulation of vascular responses by its agonists in GRK2 overexpressing mice^24^. These reports suggest that SBP elevation in the present study could result from desensitization of β-AR signaling in VSM of GRK2 overexpressing mice post epinephrine administration.

Previous studies have demonstrated that lymphocytes obtained from hypertensive patients, as well as aortas of spontaneously hypertensive rats had significantly upregulated GRK2 protein expression and impaired β-AR responsiveness^25–27^. Other studies have reported a significant attenuation of DBP lowering in *Grk2* transgenic mice at isoproterenol doses that are too low to affect HR^28^. While these results support our findings, it is important to highlight that the difference between the earlier experiments on transgenic mice and our study is the utilization of epinephrine, an endogenous β-AR agonist instead of isoproterenol, and our employment of AAV for GRK2 overexpression in order to eliminate its embryonic effects. Heart failure is an adult disease, and we assessed the short-term effects by overexpressing GRK2 in adulthood using AAV for GRK2 overexpression.

The surrogate markers of HF, including increased lung weight and ventricular *Nppb* expression were observed post GRK2 overexpression in VSM accompanied by inappropriate elevation of BP that was consequent to desensitization of β-AR signaling post epinephrine administration. These findings mimic the pathological condition associated with CS1, thus suggesting that GRK2 activity in VSM may play a role in CS1 pathogenesis.

While a few observational studies have reported elevation of GRK2 expression in human peripheral lymphocytes in HF, absolute clinical confirmation of the same in the vasculature of HF patients is not available^29,30^. William et al. reported an increase in vascular GRK2 protein expression in rat aorta with age^31,32^. Additionally, certain interventional studies have demonstrated reduced sensitivity of vascular β-AR in elderly patients and speculated on the possibility that reduced β-AR responses in vasculature might be a part of the aging process^33,34^. Given that HF, including CS1, is more prevalent in elderly patients, it is indeed possible that upregulation of GRK2 and downregulation of β-AR signaling in the vasculature of patients with CS1 are molecular features of the disease.

The present study had following limitations. First, the approach with intraperitoneal AAV injection might lead to a highly variable expression, and in the present study, some statistical work was done for obtaining results overcoming variability. Second, although ex vivo isolated vessel preparation was a well-established and widely used method as shown by Eckhart et al.^28^, we selected the blood pressure measurement rather than the ex vivo experiments, because our hypothesis was that upregulation of vascular GRK2 lead to pathological conditions similar to CS1. Third, although cAMP is a molecule immediately downstream of β-AR as shown by Eckhart et al.^28^, we could not assess cAMP levels and we alternatively assessed pMLC2 downstream of PKA. Fourth, although β-AR desensitization can be directly tested by measuring functional β-AR with radioligand binding assay or proximity ligation/biotinylation assay, we could not also assess β-AR desensitization directly and we alternatively assessed accumulation of pMLC2.

## Conclusions

Enhanced expression of GRK2 in VSM may result in inappropriate SBP elevation and other cardiovascular changes, thus leading to HF as seen in CS1.

## Methods

### Construction of recombinant AAV vectors

Recombinant AAV vectors that expressed, under the influence of the *Myh11* promoter, either the GRK2 or the GFP that served as the infection control were generated. We selected AAV-DJ8 as a virus serotype in this study after the vasculature expression trial of GFP with *Myh11* promotor. The *Grk2* gene and *Myh11* promoter were cloned from a wildtype C57BL/6 J mouse that was purchased from Clea Japan (Tokyo, Japan). The GFP-expressing plasmid was procured from Cell Biolabs (AAV-400, San Diego, California, United States). For pAAV-DJ8-GRK2 or pAAV-DJ8-GFP plasmid construction, pAAV-MCS were purchased from Cell Biolabs (VPK-410). After inserting *Grk2* or GFP and exchanging CMV promotor to *Myh11* promotor in pAAV-MCS, it was transfected into human embryonic kidney 293 cells together with pAAV-DJ8 and pHelper purchased from Cell Biolabs (VPK-420-DJ-8, 3402-02) to produce AAV-DJ8-GRK2 or AAV-DJ8-GFP vector. The vector was then purified, and concentrated by two cycles through the Amicon Ultra-15 centrifugal filters (UFC910024; Merck Millipore, Burlington, Massachusetts, United States). The purified viral preparations were stored in phosphate-buffered saline, and physical particle titers were determined using competitive PCR. The primers used for cloning *Grk2* gene and *Myh11* promoter are listed in S1 Materials and Methods.

### Experimental animals

All animal experiments were conducted in accordance with the Guide for the Care and Use of Laboratory Animals published by the US National Institutes of Health 1996. All experiments were conducted under ethical approval of the Institutional Animal Care and Use Committee of Nara Medical University (experimental number 345-4), and reported in accordance with the ARRIVE guidelines. Adult male (8-week-old) C57BL/6 J mice were purchased from Clea, Japan and intraperitoneally injected with 1 × 10^12^ vg AAV for either GRK2 (GRK2 group) or GFP (control group) overexpression under the control of the *Myh11* promoter to generate a mouse of VSM-specific GRK2 overexpression. Mice were maintained on a 12-h light/dark cycle from 08:00 to 20:00 with unrestricted access to food and water and were analyzed on reaching 16 weeks of age.

### Experimental protocols

Anesthesia was induced in all groups of mice by inhalation of 2% isoflurane (Isoflurane Inhalation Solution; Mylan, South Point, Pennsylvania, United States) in room air until loss of the pedal withdrawal reflex. The surgical plane of anesthesia was maintained by delivery of 2% isoflurane in room air via a nose cone, while maintaining a body temperature of 38.0 °C with a heat pad (2-way heater; GEX, Osaka, Japan). Mice were subsequently subjected to invasive BP analysis or echocardiography. The former involved cannulation of the right carotid artery with a 1.4 F catheter with pressure sensors (SPR 671; Millar Instruments, Houston, Texas, United States) along with continual monitoring of BP. All parameters were digitized and recorded using an analog-to-digital converter (PowerLab; ADInstruments, Dunedin, New Zealand). BP signals were used to derive SBP, MAP, DBP, and HR with the help of a pressure laboratory chart software (LabChart version 5; ADInstruments). Post completion of surgical preparation, the BP monitoring plane was maintained by delivery of 1% isoflurane in room air via a nose cone, and a stabilization period of more than 5 min was allowed before commencement of basal BP recording. The changes in BP and HR in response to intraperitoneal injection of 1 mg/kg epinephrine (324900; Merck Millipore) diluted in 100 μL saline or the same volume of saline were subsequently determined. The maximal change in BP from baseline was quantified approximately 5 min post injection of epinephrine or saline. This was followed by administration of 2% isoflurane in room air and dissection of mice to excise tissues within a few minutes. After the dissection of mice, firstly, the weights of the lungs were recorded. Secondly, the weights of the heart were recorded. And finally, other excised tissues were either immersed in phosphate buffer supplemented with 10% formalin or frozen. In experiments involving echocardiography, the procedure was performed before and after administration of 1 mg/kg epinephrine. Mice were dissected 15 min post epinephrine injection, followed by measurement of *Nppb* mRNA expression levels. All other processes were identical to those followed during invasive BP analysis.

### Echocardiography

Mice were imaged at 16 weeks of age for echocardiographic analysis. Towards this end, mice were initially anesthetized in a chamber containing 2% isoflurane in room air and subsequently placed on a heating pad at 37 °C under maintenance anesthesia (1% isoflurane in room air). Echocardiographic assessment was then performed using Prospect ultrasound (S-Sharp, New Taipei, Taiwan) with a real-time microvisualization transducer (Prospect Probe, 40 MHz at a rate of 30 frames/s) that was applied parasternally to the shaved chest wall. Images were acquired in the parasternal long-axis, parasternal short-axis, and short-axis M-mode (SAMM). SAMM images were acquired by placing the M-mode cursor vertically in the parasternal short-axis view when both papillary muscles were visualized. Images and data were exported for offline calculations using the Prospect software (Prospect version 1.0.5), and SAMM images were used to measure LVEF. Echocardiographic parameters were not normalized to body weight.

### Assessment of pulmonary edema

Evaluation of pulmonary edema was based on the quantity of pulmonary fluid retention. Wet lung weights were determined by weighing the bilateral lungs immediately post each individual excision. Dry lung weights were assessed after drying the organs at 60 °C for 5 days.

### RNA isolation and quantitative PCR

Total RNA was extracted from the brain, heart, liver, kidney, and aorta using TRIzol reagent (15596018; ThermoFisher, Waltham, Massachusetts, United States) and an RNeasy Mini Kit (74104; QIAGEN, Venlo, Netherlands). A total of 1000 ng of RNA was reverse transcribed using the PrimeScript RT Master Mix (RR036A; TAKARA Bio, Shiga, Japan), and real-time PCR was performed in duplicate using Fast SYBR Green Master Mix (4385612; Thermo Fisher) with specific primers for the target sequences. The primers used for PCR are listed in S1 Materials and Methods.

### Immunohistochemistry of GRK2

Aorta samples were washed with phosphate-buffered saline (PBS, pH 7.4) prior to preparation of two cross sections during dissection. One cross-section was positioned with the luminal side facing downwards on a cup (Cryomold; Sakura Finetek, Torrance, California, United States) and covered with the Tissue-Tek optimal cutting temperature compound (O.C.T. Compound; Sakura Finetek). The aortic sample was placed on dry ice for at least 5 min, and frozen samples were stored at − 80 °C until sectioning was performed. The remaining cross-sections were fixed in 4% paraformaldehyde, dehydrated, and embedded in paraffin prior to GRK2 immunohistochemical analysis. This was accomplished by sectioning tissue samples into 4-µm thick slices that were made to adhere to glass slides. GRK2 was detected in these sections using an anti-GRK2 rabbit monoclonal antibody (1:50, ST05-60; Novus, Littleton, Colorado, United States). Staining was visualized with the aid of horseradish peroxidase-conjugated secondary antibodies and 3,3’-diaminobenzidine (DAB; Nichirei Biosciences, Tokyo, Japan). Slides were counterstained with Harris modified hematoxylin, and subsequently dehydrated using ethanol solutions of incremental concentrations and xylene before being mounted under coverslips using PARA mount-N (Meiji Seika Pharma, Tokyo, Japan).

### Assessment of desensitization of vascular β-AR signaling using phospho-myosin light chain 2/αSMA

For quantitative histological assessment of desensitization of vascular β-AR signaling, pMLC2, a marker of vasoconstriction, and αSMA, a VSM cell marker, were quantified by immunofluorescent staining using the VECTOR M.O.M. Immunodetection Kit (#PK-2200, #FMK-2201; Vector Laboratories, Burlingame, California, United States) along with a BZ-X710 microscope and its associated analyzer software system (BZ-X710 system; Keyence, Osaka, Japan). Briefly, aortic samples were sectioned into 4-µm thick slices in a cryostat at − 20 °C and made to adhere to glass slides. These sections were air-dried in a dryer for 30 min at room temperature, followed by fixation in acetone at room temperature for 10 min. Both primary antibodies, namely anti-phospho-myosin light chain 2 rabbit polyclonal antibody (3671; Cell Signaling Technology, Danvers, Massachusetts, United States) and anti-αSMA (48938; Cell Signaling Technology) were used at a working dilution of 1:200. TEXAS RED AVIDIN DCS and WGA-Alexa Fluor 488 (1:1000, A11008; Thermo Fisher) were used for visualization of αSMA and pMLC2, respectively, and cell nuclei were stained with DAPI. The pMLC2-positive areas were estimated as percentages of the αSMA-positive area using the BZ-X710 system. The αSMA-positive and pMLC2-positive areas in Fig. 5C have been depicted in red and green, respectively. The green regions represent pMLC2 fluorescence as well as autofluorescence emitted from the elastic fibers and interstitial tissue, and yellow regions in the merged images are truly representative of pMLC2-positive areas. Yellow regions divided by red regions equal pMLC2 Positive Area (% αSMA Positive Area).

### Statistical analysis

Continuous variables are expressed as mean ± standard error of the mean (SEM) or median (25th–75th percentile) and categorical variables as numbers and percentages. Differences between groups were tested using the unpaired Student’s *t* test, Mann–Whitney *U* test, Pearson’s chi-square analysis, Fisher’s test, or two-way ANOVA, as appropriate. A two-tailed P value of less than 0.05 was considered to be indicative of statistical significance. All statistical analyses were performed using the R software (University of Auckland, Auckland, New Zealand).

## Supplementary Information

Supplementary Information.

## Data Availability

The datasets generated and analyzed during the current study are available from the corresponding author on reasonable request.
