# Supplementary material for: Overexpression of GRK2 in vascular smooth muscle leads to inappropriate hypertension and acute heart failure as in clinical scenario 1
Source: Sci Rep. 2023 May 12;13:7707. doi: 10.1038/s41598-023-34209-5 (PMC10182096; doi:10.1038/s41598-023-34209-5)
Supplement: Supplementary file 1 — Supplementary Information. [file 41598_2023_34209_MOESM1_ESM.docx]

Supplemental Material

**Overexpression of GRK2 in vascular smooth muscle leads to inappropriate hypertension and acute heart failure as in Clinical Scenario 1**

Hiroki Yano, MD^1^, Kenji Onoue, MD, PhD^1^, Shiho Tokinaga^1^, Tomoko Ioka^1^, Satomi Ishihara, MD, PhD^1^, Yukihiro Hashimoto, MD^1^, Yasuki Nakada, MD, PhD^1^, Hitoshi Nakagawa, MD, PhD^1^, Tomoya Ueda, MD, PhD^1^, Ayako Seno, MD, PhD^1^, Taku Nishida, MD, PhD^1^, Makoto Watanabe, MD, PhD^1^, Yoshihiko Saito, MD, PhD^1, *^

^1^Department of Cardiovascular Medicine, Nara Medical University, Kashihara, Nara, Japan

Short title: Vascular GRK2 and CS1 like acute heart failure

Corresponding author: Yoshihiko Saito, MD, PhD, Department of Cardiovascular Medicine, Nara Medical University, 840 Shijocho, Kashihara 634-8522, Japan. Tel: +81(0744)22-3051, Fax: +81(0744)22-4121, E-mail: [yssaito@naramed-u.ac.jp](mailto:yssaito@naramed-u.ac.jp)

**S1 Materials and Methods**

***Primers of cloning and quantitative polymerase chain reaction***

The polymerase chain reaction (PCR) forward and reverse primer sequences used for cloning of each gene were as follows: *Grk2* CGCCGGAATTCATGCAGAAGTATCTGGAGGACCG and ATAATGTCGACTCAGAGGCCGTTGGCACTGCCAC, *Myh11* CGCCACGCGTAGCCCAGTGTCTGGGCATTT and CCTGTGGTGGGTGGGTCTCAGGCGCCTATA. EcoR Ⅰ and Sal Ⅰ digestions of *Grk2* cDNA were used for cloning with 5′ and 3′ probes, respectively. Mlu Ⅰ and Sac Ⅱ digestions of *Myh11* cDNA were used for cloning with 5′ and 3′ probes, respectively.

The real-time PCR external calibration curve method using *18S* ribosomal RNA for normalization was employed to estimate the quantity of target mRNA in the samples. The real-time PCR forward and reverse primer sequences used for each gene were as follows: *Grk2* GCGCATCAAGATGAAGCAGG and CGTCCAGAAGGATGTTGGCT, *Mlc2* AAGAAGCGGATAGAAGGCGG and TCGTCCTAGGGCAGCAAATG, *Nppb* CTGAAGGTGCTGTCCCAGATGATTC and CTGCATCTTGAATTGCTCTGGAGAC, and *18S* ribosome GTCTGTGATGCCCTTAGATG and AGCTTATGACCCGCACTTAC.

**
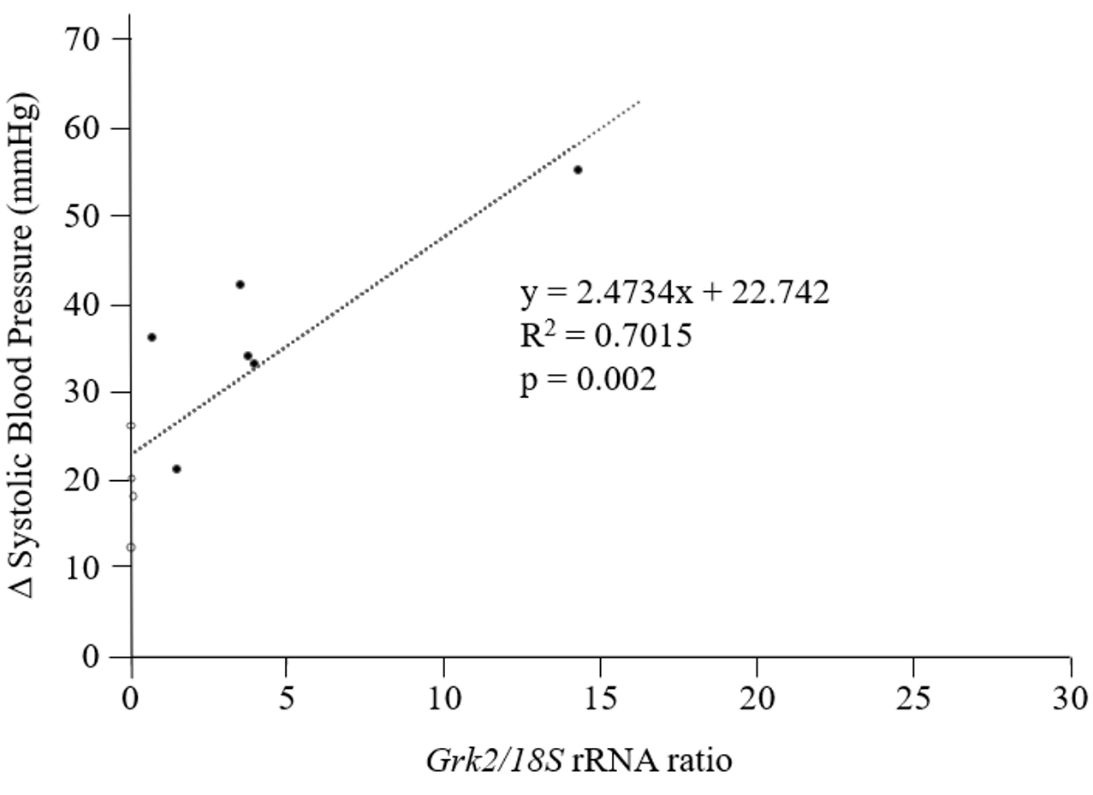
**

**Supplementary Figure S1** The relationship between *Grk2*/*18S* rRNA (horizontal axis) and SBP changes post epinephrine injection (vertical axis) in 10 animals. The regression line (dotted line) and equations have been depicted. White circle - 4 control mice; black circle **-** 6 GRK2 mice. GRK2: G protein-coupled receptor kinase 2; rRNA: ribosomal RNA; SBP: systolic blood pressure.


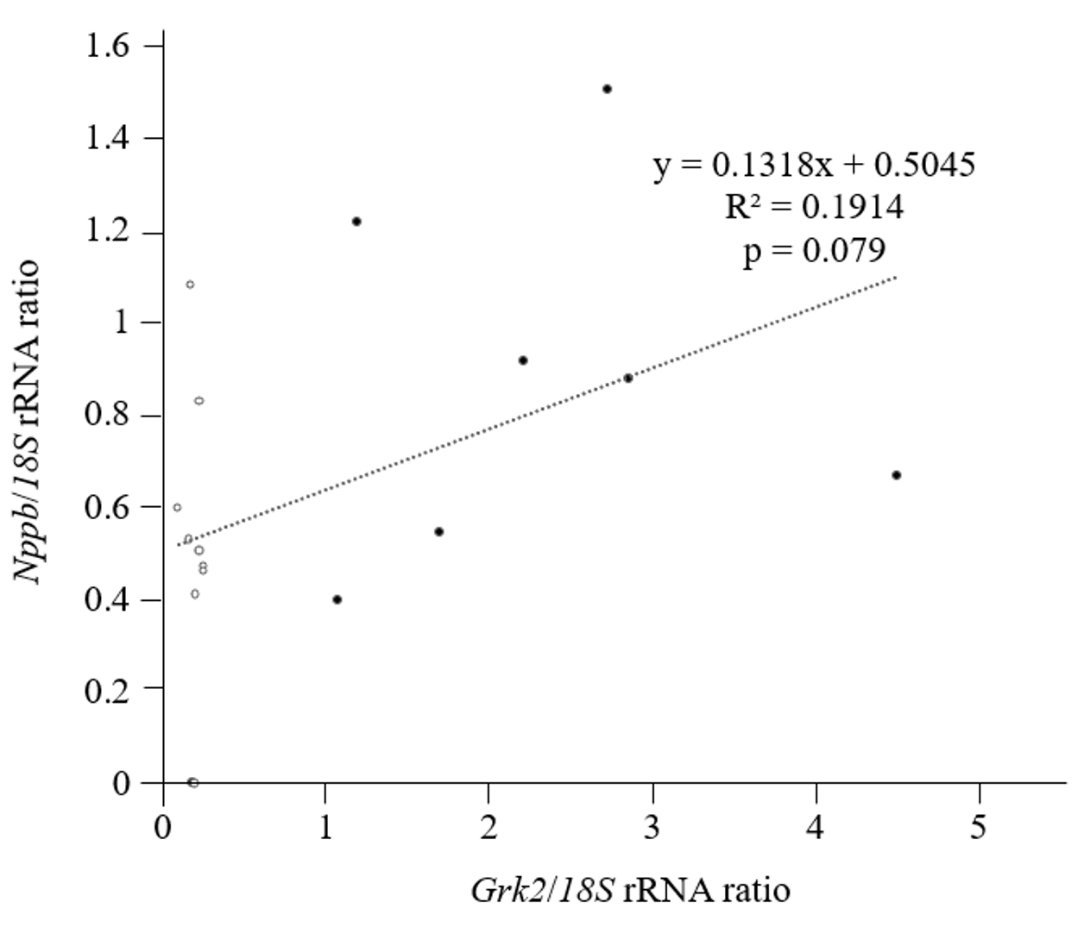


**Supplementary Figure S2** The relationship between *Grk2*/*18S* rRNA (horizontal axis) and *Nppb*/*18S* rRNA post epinephrine injection (vertical axis) in 17 animals. The regression line (dotted line) and equations have been depicted. White circle - 10 control mice; black circle **-** 7 GRK2 mice. *Nppb*: *natriuretic peptide B*; GRK2: G protein-coupled receptor kinase 2; rRNA: ribosomal RNA.
